# Effects of the COVID-19 Pandemic on the Performance of Marathon Runners

**DOI:** 10.1155/tsm2/9969371

**Published:** 2025-06-04

**Authors:** Javier Lluch, Félix Martínez-Giménez, Francisco Abad, Javier Garrido Martínez

**Affiliations:** ^1^Automatics and Industrial Informatics Research Institute, Polytechnic University of Valencia, Valencia, Spain; ^2^Pure and Applied Mathematics University Research Institute, Polytechnic University of Valencia, Valencia, Spain

**Keywords:** athlete performance, COVID-19, marathon, pace behavior

## Abstract

Analyzing the performance of marathon runners is a study of vital importance for optimizing athletes' results. The COVID-19 pandemic and its measures against its spread resulted in a drastic change in the way of life of most of the population, including athletes, who saw their training habits modified, in addition to the possible short-, medium-, and long-term consequences that infection with the new virus could cause in their health. This study analyzes through normality analysis, Kolmogorov–Smirnov, Chi-square tests, and the Wasserstein distance the finish times and paces of more than 900k athletes (filtered by age range and gender) in major marathons in different cities around the world to determine the effects of the pandemic on their performance. The analysis using the Wasserstein distance shows that the period of years with the most significant differences in race pace was 2019–2021 (pre- and postpandemic years) in practically all the marathons analyzed, while the analysis using chi-square shows differences in that period of years in some cities, age, and gender groups. We found significant differences between 2019 and 2021 in pace behavior in some age and gender groups, shown by the Wasserstein distance and chi-square test.

## 1. Background

The COVID-19 pandemic, a global health crisis that emerged in late 2019, has led to unparalleled disruptions across various aspects of society. Among the many facets of life affected, the world of sports has experienced profound challenges and transformations. Marathon running, a sport demanding unwavering dedication and precise preparation, has not been immune to the pandemic's ramifications. Athletes worldwide have grappled with uncertainties, race cancellations, and adaptations to their training regimens, all of which have the potential to influence their performance in unforeseen ways [1, 2].

This article seeks to explore the multifaceted impacts of the COVID-19 pandemic on marathon athlete performance, focusing on race finishing times and athletes' pace as key metrics. Our comprehensive analysis draws from a substantial dataset encompassing over 900,000 athletes from major worldwide marathons in 2018, 2019, 2021, 2022, and 2023, classified by age range and gender.

The justification for this study is rooted in the compelling need to understand the consequences of the COVID-19 pandemic on marathon athletes on a global scale. Several factors highlight the significance of this research.

### 1.1. Objectives of the Study

• *Pace analysis*: we will examine the race finishing times and race pace of marathon athletes from various major marathons, investigating trends, variations, and shifts in performance levels.
• *Health impact*: using the pace behavior metric introduced by Deaner et al. [3] and employing chi-square tests and the Wasserstein distance, we aim to determine whether the pandemic has had a statistically significant impact on pace behavior among marathon runners, which could indicate an effect on the cardiorespiratory system.

Numerous studies have analyzed the impact of COVID-19 on human health, as shown in [4–8]. These studies range from epidemiological [9, 10] and immunological [11] analyses to the consequences of the disease on the cardiorespiratory system like in Long et al. [12]; Puntmann et al. [13]; Lindner et al. [14]; and Siripanthong et al. [15]. In the particular case for our study, focusing on athletes, we have found some studies that analyze the consequences of the pandemic for runners of different disciplines. For example, in Chan et al. [2], the authors examined the training routines of runners using data collected from their wearable devices. They assessed the frequency, intensity, and duration of workouts. They tracked weekly mileage and location of sports activities for 9 months, both before and after COVID-19, when restrictions were put in place in March 2020. The findings revealed an increase of 3 km per week in the distance runners ran after the beginning of restrictions, as well as an increase of 0.3 additional weekly training sessions. However, no changes were noted in the intensity or duration of each session. These results contrast with the findings of Afonseca et al. [1], who analyzed data from more than 36,000 athletes in greater temporal detail. They concluded that at the beginning and during much of the pandemic, the number of athletes, distance run, and training time decreased, while only in the second quarter and at the end of 2020 did they increase to 2019 levels. These pandemic-induced changes in training could affect athlete performance during the marathon. None of these studies examine differences in athlete pacing during marathons between years or with samples as large as those in our study.

In the study by Sriram et al. [16], the authors analyze the performance of runners in the world's busiest 10K race (Peachtree 10 km Road Race). Participants' residence and pace were correlated with publicly available durations of confinements, facemask mandates, and gym closures, plus COVID-19 hospitalization incidence and per capita mortality from March 2020 to March 2021, while controlling for age and gender. Pandemic-era runners improved performance compared with prepandemic times, consistent across all age and gender groups, with the greatest benefit in younger people. COVID-19 mitigation restrictions and disease severity did not affect performance, suggesting that unmeasured lifestyle changes (increased time for training due to restrictions) may have contributed to improved fitness. This study does not analyze the effects of the pandemic on marathons in postpandemic years.

In the study by Scheer et al. [17], the authors examined the effects of the COVID-19 pandemic on endurance running and ultraendurance running and analyzed goals and events during the COVID-19 pandemic (observation period March 2020–October 2020) relative to the same period before the COVID-19 outbreak (March 2019–October 2019). Mean marathon completion times decreased during the pandemic in both men and women. In this case, only race times from the prepandemic period and times from the few races that could be held during the pandemic period were analyzed. The pace was not examined.

In addition, we found a study directly analyzing the health effects of COVID-19 in runners who had contracted the disease. Barker-Davies et al. discussed the case of an elite marathon runner over 30 years old who suffered from chest pains after contracting COVID-19 [18]. His subjective tolerance to sport decreased (he describes it as the inability to accelerate quickly). He underwent a cardiopulmonary exercise testing (CPET) 5 months after contracting COVID-19 and was compared with another CPET performed 15 months before the disease. Although supranormal maximal oxygen consumption was maintained, the anaerobic threshold (AT), a better predictor of endurance performance, was reduced from 84% to 71% of the predicted peak VO2 max. Likewise, fat oxidation at AT was decreased by 21%. The reduction in AT suggests a peripheral impairment of aerobic metabolism. This finding may result from virus-mediated mitochondrial dysfunction, beyond normal “deconditioning,” associated with impaired fat oxidation.

These studies suggest that there may be significant differences in the behavior of runners before and after a global pandemic due to a respiratory virus like COVID-19.

Menting et al. [19] showed how the development of pacing behavior begins in childhood and evolves positively with age during adolescence and training. This positive development continues until the age of 35, according to Tanaka and Seals [20], when there are modest declines until 50–60 years and continues to decline progressively from that age. Cuk et al. [21] and Zavorsky et al. [22] also analyzed these changes in pacing behavior across age and between genders in 10 km and marathon runners.

In the studies by Bonanad et al. [23], Romero Starke et al. [24], Manry et al. [25], Zhang et al. [26], and Henkens et al. [27], the epidemiological differences of coronavirus with respect to age, gender, and certain previous ailments are shown. These studies conclude that there is a higher risk of developing severe forms of COVID-19 in patients with previous pathologies and older age. Gender differences (primarily in men) could also affect the severity of COVID-19.

All these studies show the importance of considering gender and age differences to determine the differences in pacing behavior in our study.

## 2. Materials and Methods

For our study, we obtained data from the official websites of each marathon, with the actual classification times. We downloaded the total race times, half marathon times, and times for every 5 km, in addition to other data of interest, such as age range and gender (male/female). We focus on the marathons held in 2018-2019 and 2021–2023 (years before and after the pandemic) in Boston, Berlin, Chicago, London, Paris, and Valencia. These races have been chosen because they have the highest number of participants and provide the largest amount of data available in the USA and Europe. In our analysis, we study differences in running pace behavior, taking into account age ranges (0–39, 40–59, and 60+) and gender (M/F) since according to multiple studies, these are indicators of significant differences both in marathon results Menting et al. [19]; Tanaka and Seals [20]; Cuk et al. [21]; Zavorsky et al. [22] and in COVID-19 severity Bonanad et al. [23]; Romero Starke et al. [24]; Manry et al. [25]; Zhang et al. [26]; Henkens et al. [27]. Since the data were extracted from multiple sources, the age ranges were chosen using the widest common ranges to compare the data offered by the different marathon classifications. Each race uses its ranges, which have been unified into the three chosen ones, which are present across all races. Creating more age ranges could result in small groups that would not be meaningful.

First, we analyzed the normality of the finishing times' data to determine whether we could apply statistical techniques to normal data.

**Figure 1:**
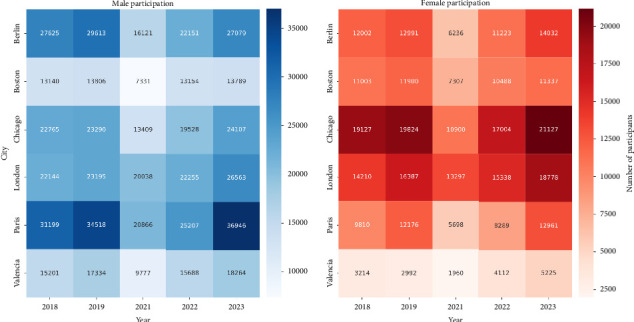
Heatmap of participation in each race by gender.

When performing the pacing study, we filtered the results to avoid anomalous data effects. Time data greater than 382.5 min (9.07 min/km) were removed because they indicate a running pace that suggests the athlete has stopped running. In addition, since we want to look at the overall trends of nonprofessional runners to analyze the trend in the general population, we eliminated times under 168 min (3.98 min/km), as these are considered elite runners who may have had access to different training during the pandemic due to their status as elite athletes, as well as having different prior physical conditioning than nonprofessional runners. After filtering the data, we have 180 groups of tuples (city–year–age range–gender).  Table 1 summarizes the number of athletes and the average finishing times for each marathon per city and year.  Figure 1 shows a heatmap graph with the evolution of participation in each race by gender.

Running pace is calculated as the units of time required to run a given unit of distance, in our case, minutes per kilometer.

(1) $$\begin{array}{c} \text{pace}=\frac{\Delta t}{\Delta d}. \end{array}$$

Next, we analyze the results of the race pace using the measure introduced by Deaner et al. in [3]. They classify the pace behaviors of the athletes into three categories according to the variation in race pace between the first half of the marathon and the second half. The categories are: “Good Pace” (GP) for those with less than 10% variation, “Mid Pace” (MP) for those with a pace variation between 10% and 30%, and “Slow Pace” (SP) for those with a pace variation greater than 30%. To perform the analysis, we compute the chi-square test to compare the distributions of runners in these three pace behavior categories between prepandemic and postpandemic years, filtering by gender and age range. In addition, we compute the Wasserstein distance between these categorical pace behavior distributions. We utilized the Wasserstein distance because, unlike other tests based on *p* values, it can compare two different years, determine if there are differences, and quantify the magnitude of these differences. This distance, also known as the earth mover's distance, measures the minimum amount of “work” required to transform one distribution into another, where “work” is quantified as the amount of distribution weight that must be moved multiplied by the distance it has to be moved Kantorovich [28]. Thus, a higher distance value indicates lower similarity.

## 3. Results

**Figure 2:**
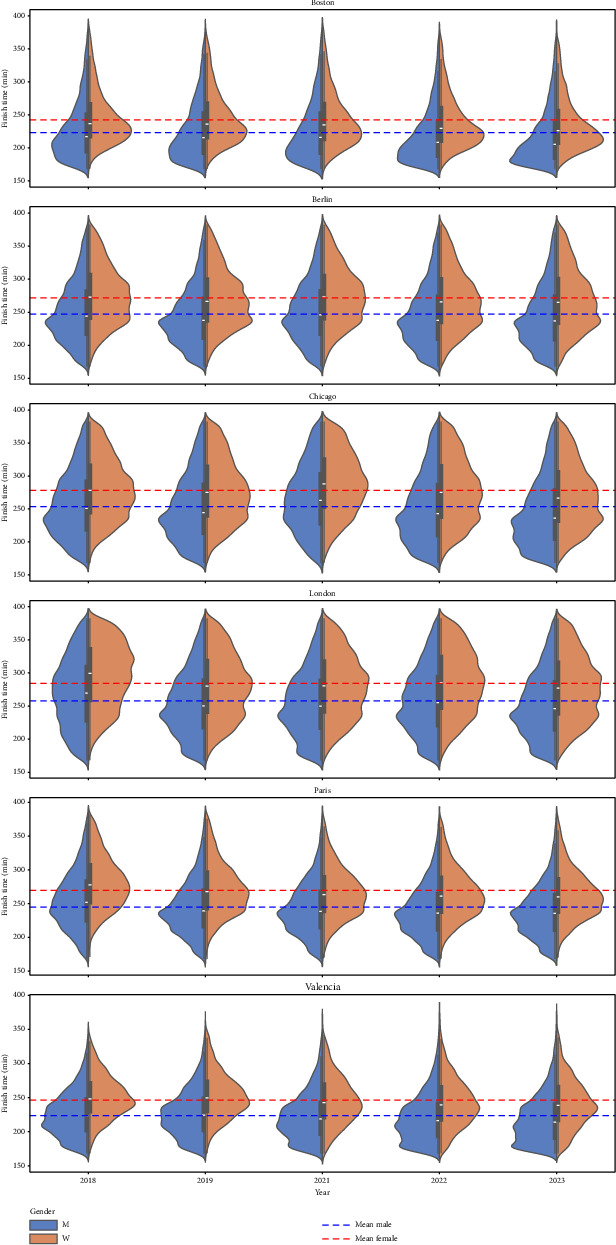
Finish time distribution of each race separated by gender. The horizontal lines show the global average finish time per gender for all the editions of a race.

**Figure 3:**
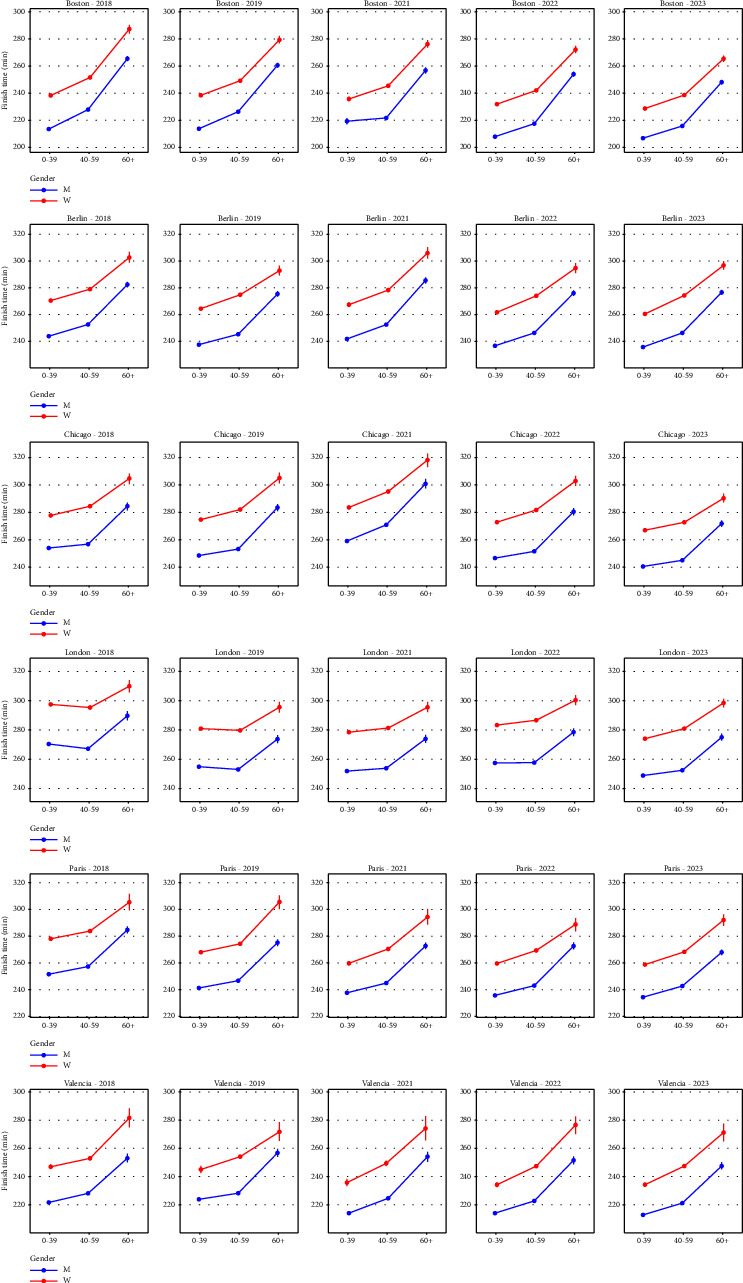
Finish time averages grouped by age and gender with 95% confidence intervals.

First, we filter the data by city (Boston, Berlin, Chicago, London, Paris, and Valencia), year (2018, 2019, 2021, 2022, and 2023), gender (male and female), and age range (0–39, 40–59, and 60+), resulting in 180 groups. The use of the variables city, gender, age, and year is justified for the following reasons. According to the testimony of marathon runners, each major marathon has its particularities due to terrain, climate, tradition, etc. These particularities make one city's marathon very different from the others. We filter by gender because it is well known, according to medical reports, that COVID-19 affects men and women differently. A similar situation occurs with the variable age. Finally, we must use the variable year to separate races pre- and postpandemic. We perform the usual Shapiro–Wilk and Anderson–Darling normality tests on each group. It turns out that using the finishing time as the variable to analyze each group, the *p* values from the normality tests are very close to 0 (all of them smaller than 0.05), indicating that finishing times in each group do not follow a normal distribution. Therefore, the usual tests to compare normal distributions' mean and standard deviation seem inappropriate, so we use nonparametric tests.  Figure 2 shows the finish time distribution for each race by gender. The horizontal lines show the average finish time by gender.  Figure 3 shows the finish time averages of each race grouped by age and gender, with 95% confidence intervals.

Next, filtering data for city, gender, and age range, we perform a two-sample Kolmogorov–Smirnov test for years 2018 and 2019, another one for years 2019 and 2021, for years 2021 and 2022, and finally for years 2022 and 2023, again using the finishing time as the variable to compare. Analyzing the results for each group when comparing 2019 and 2021, we can conclude that the two distributions are different, indicating that each group performed differently in 2019 and 2021. Unfortunately, the same conclusion can be derived when comparing prepandemic years 2018 and 2019 and postpandemic years 2021 and 2022.

The above discussion drives us to the conclusion that the variable total finishing time is too fine-grained and probably catches differences in the performance of marathon runners when comparing any two different years.

At this point, we decide to use a categorical variable to look for the effects of COVID-19 on the performance of marathon runners. Due to its acceptance in the literature, we use the variable “pace behavior” defined by Deaner et al. [3]. For each runner, we compute their pace behavior with the following formula:

(2) $$\begin{array}{c} \text{Pace behavior}\left(\text{runner}\right)=\left\{\begin{array}{c} \text{GP},\text{ if }\frac{{HM}_{2}-{HM}_{1}}{{HM}_{1}}<10\%,\\ \text{MP},\text{ if }10\%\leq \frac{{HM}_{2}-{HM}_{1}}{{HM}_{1}}\leq 30\%,\\ \text{SP},\text{ if }30\%<\frac{{HM}_{2}-{HM}_{1}}{{HM}_{1}}. \end{array}\right. \end{array}$$

In the formula for calculating pace behavior, *HM*_1_ and *HM*_2_ are the time in minutes of the first and second half marathon for runner, respectively.

Next, for each of the 180 groups, we compare the percentage of runners having GP, MP, or SP in that group. As an example, the percentages of runners having GP, MP, and SP in the 12 groups corresponding to males in the Boston marathon are shown in Table 2:

To analyze pacing behavior, we used the formula Deaner et al. [3] described to classify running paces between runners and determine if significant differences existed between years. Subsequently, we conducted the Chi-square test between statistical distributions to identify differences in the ratio of categorical paces between years, aiming to ascertain if there were greater disparities between prepandemic and postpandemic years. In our case, the null hypothesis is that both distributions have similar proportions of pace behavior, while the alternative hypothesis suggests significant differences in those proportions. We selected a significance level of 5% (values lower than the significance level indicate rejection of the null hypothesis). The test results with *p* values less than the significance level are presented in Table 3.

As we can see, significant differences exist between 2019 and 2021 in the Berlin and Chicago marathons (in all age ranges and genders) and in the London marathon for females older than 60 years.

In addition to the chi-square test, we conducted studies with different distances and statistics to analyze differences in the pace behavior over the years. We use Wasserstein distance, whose higher distance value indicates lower similarity. We selected a city and calculated the Wasserstein distance between 2018 and 2019, 2019 and 2021, 2021 and 2022, and finally, 2022 and 2023, filtering by age range and gender. The results are presented in Table 4.

As seen in Table 4, the pair of years 2019–2021 or 2021–2022 (pre and post of 2021) gets the maximum distance in 15 out of 36 groups analyzed. On the other hand, the pair of years 2018–2019 or 2022–2023 (further from year 2021) gets the minimum distance in 23 out of the 36 groups. This indicates that the pandemic probably had some effect, whose aftermaths affected the performance of marathon runners in the year 2021.

The striking London and Paris results show the largest distance in almost all age groups and genders between 2018 and 2019. In the case of London, these results could be explained by the fact that London 2018 was the marathon with the highest temperature of the whole series in that city1. If we remove the 2018-2019 distances, we observe that all 3 female groups have the maximum distance between years 2019–2021 and between years 2022–2023 for men. For the case of Paris, we found that 2018 had the hottest marathon day in 2018–2023^2^ with a min/max temperature of 11°/22° and no wind. Compared with the rest of the editions, 1°/8° in 2019, 6°/16° in 2021, 1°/8° in 2022, and 8°/9° in 2023. As we did before, if we remove distances for 2018–2019, we observe that the maximum in 5 of the 6 groups is reached in 2022-2023. This fact could indicate that maybe for runners of the marathons in those cities, the effects of the pandemics lasted longer than in the other cases. But to jump to that conclusion, a deeper analysis should be made of the marathons in London and Paris.

**Figure 4:**
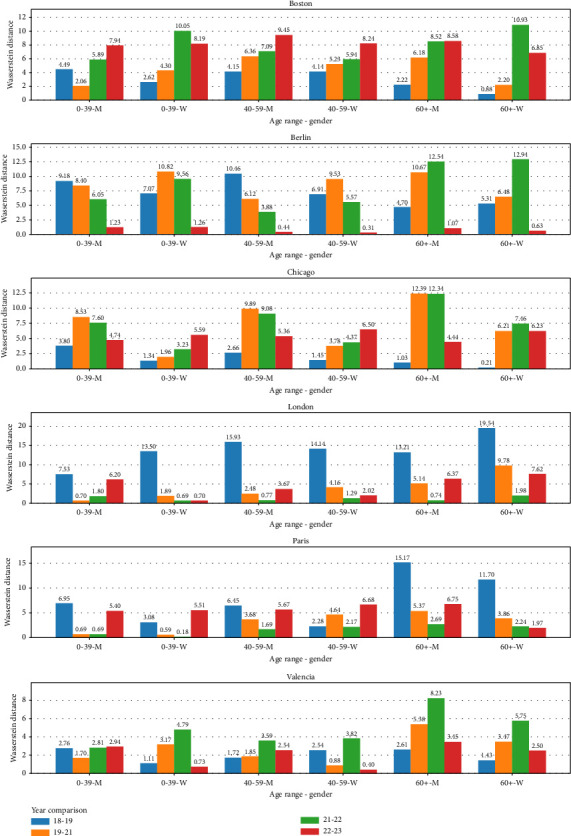
Wasserstein distance ranking results (same data as Table 5).

Then, we analyzed those groups where the distance was maximum for 2019–2021 and 2021–2022; one was the maximum among the rest of the years and the other had the second greatest distance. There were 9 out of 36 groups, shown at the top in Table 5. Finally, we analyzed groups where years 2019–2021 and 2021-2022 were between the three higher distances. There were 23 out of 36 groups, as shown in Table 5 and graphically in Figure 4.

**Figure 5:**
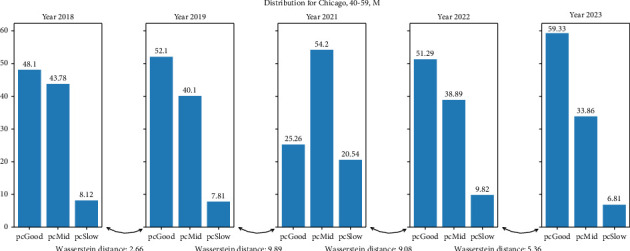
Percentages of pace behavior distributions for the city of Chicago, age 40–59, males. Wasserstein distance from one distribution to another is shown underneath.

**Figure 6:**
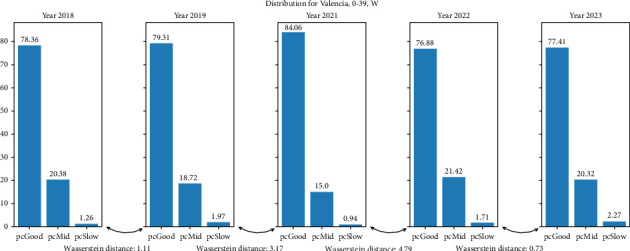
Percentages of pace behavior distributions for the city of Valencia, age 0–39, females.

In Figure 5, we plot the distribution of pace behavior of Chicago, 40–59, M (where the Wasserstein distance is maximum between 2019–2021). We can see how, between the distributions from 2019 to 2021, there is a change in the proportion of GP toward MP and SP. This same effect occurs in the five groups where the distance is maximum for 2019 and 2021. When we analyze distributions where the two higher Wasserstein distances are 2019–2021 and 2021-2022, we find that most of these distributions follow the above rule (6 out of 9, the only groups not following this rule are from Valencia). In Figure 6, we plot Valencia, 0–39, W. Analyzing the distributions where the three higher Wasserstein distances are 2019–2021, 2021-2022, and any other, we find that the groups that follow the above rule are 13 out of 23; the only groups not following this rule were those from Boston and Valencia (5 groups each).

## 4. Discussion

The goal of this study was to determine whether marathon runners' pace performances differed between the prepandemic (2018-2019) and postpandemic (2021–2023) periods, considering age ranges (0–39, 40–59, and 60+), gender, and six major city marathons (Boston, Berlin, Chicago, London, Paris, and Valencia). Our findings reveal that the COVID-19 pandemic was indeed associated with quantifiable shifts in runners' marathon performance, specifically in their pacing behavior. This section discusses these results in the context of previous research, potential confounding factors, and avenues for further investigation.

Normality tests (Shapiro–Wilk and Anderson–Darling) on finishing times within age and gender groups consistently rejected the normality assumption. This underscores that a direct comparison of yearly finishing times with parametric tests would be inappropriate.

Comparisons of finishing-time distributions (e.g., 2018 vs. 2019, 2019 vs. 2021, and 2021 vs. 2022) suggested that every pair of consecutive years appeared significantly different. Although valid statistically, this result was less practically informative since marathons can differ yearly due to course changes, weather, and participant profiles.

To mitigate the high sensitivity of finishing-time comparisons, we adopted the “good,” “mid,” and “slow” pacing categories proposed by Deaner et al. [3]. By grouping runners according to the percentage drop in pace between the first and second half of the race, we obtained more robust comparisons of pacing strategy rather than raw finishing times.

The Wasserstein (earth-mover's) distance allowed us to quantify the magnitude of differences between pacing distributions from one year to another.

Our approach and findings thus offer a roadmap for future research on how global-scale disruptions impact endurance sports and highlight the importance of pace-based evaluations in understanding population-level changes in athletic performance.

## 5. Conclusions

Our comprehensive analysis of more than 900k marathon athletes' pace behavior before and after the COVID-19 pandemic reveals significant shifts in performance dynamics. We found that finishing times do not follow a normal distribution and cannot be analyzed using the Kolmogorov–Smirnov test for differences between these years. By categorizing pace into three groups—good, mid, and slow—we applied the chi-squared test and Wasserstein distance to assess variations across the years 2018-2019, 2019–2021, 2021-2022, and 2022-2023.

Our findings indicate differences in pace behavior between 2019–2021, particularly noticeable in specific cities, age ranges, and among different genders (Berlin, Chicago, and London), as determined by the Chi-squared test.

Furthermore, the Wasserstein distance corroborates these observations, highlighting the differences in pace behavior between 2019–2021 and 2021-2022 across all examined demographics.

Our results are a stark reminder of the pandemic's profound influence on athletic performance, mirroring the broader societal and lifestyle changes during this period. This significant finding warrants further exploration into the underlying causes of these shifts and potential strategies to aid athletes in reclaiming their prepandemic performance levels.

## Figures and Tables

**Table 1:** Dataset summary.

City	Year	Count	Finishing times (min)		
			Mean	Std	Median
Boston	2018	24,143	236.26	43.15	227.9
Boston	2019	25,706	235.46	43.42	227.1
Boston	2021	14,638	235.59	44.03	227
Boston	2022	23,642	228.44	41.95	219.65
Boston	2023	25,126	226.05	41.94	216.4

Berlin	2018	39,627	258.86	46.68	253.7
Berlin	2019	42,604	252.47	45.59	245.6
Berlin	2021	22,357	258.25	46.6	254.3
Berlin	2022	33,374	253.38	47.49	247
Berlin	2023	41,111	253.14	48.16	246

Chicago	2018	41,892	268.02	49.61	264.6
Chicago	2019	43,114	264.52	51.06	259.7
Chicago	2021	24,309	276.51	50.81	275.1
Chicago	2022	36,532	263.7	52.3	259
Chicago	2023	45,234	256.91	51.65	250.2

London	2018	36,354	280.32	52.6	281.5
London	2019	39,582	265.69	50.58	263
London	2021	33,335	264.79	50.67	262.3
London	2022	37,593	269.84	51.36	267.6
London	2023	45,341	263.08	50.89	259

Paris	2018	41,009	261.55	43.17	258.3
Paris	2019	46,694	252.1	41.51	247.1
Paris	2021	26,564	247.94	39.65	243.5
Paris	2022	33,496	246.38	40.31	241.2
Paris	2023	49,907	245.67	39.56	241.2

Valencia	2018	18,415	230.89	34.25	228.3
Valencia	2019	20,326	231.25	35.23	228.6
Valencia	2021	11,737	226.74	35.6	223.1
Valencia	2022	19,800	225.61	36.47	221.9
Valencia	2023	23,489	224.32	36.87	220.4

All	All	967,051	253.45	48.39	246.8

**Table 2:** Percentages of good, mid, and slow paces for males in the Boston Marathon.

Year	Age range	GP (%)	MP (%)	SP (%)	Count
2018	0–39	46.82	42.5	10.69	4210
2018	40–59	40.5	50.71	8.78	7393
2018	60+	25.5	59.98	14.51	1537

2019	0–39	35.76	48.35	15.9	4385
2019	40–59	34.28	52.24	13.47	7689
2019	60+	22.17	60.8	17.03	1732

2021	0–39	38.84	46.91	14.25	1761
2021	40–59	43.81	44.31	11.875	4320
2021	60+	31.44	52.32	16.24	1250

2022	0–39	55.74	36.69	7.57	3938
2022	40–59	54.94	39.41	5.66	7425
2022	60+	44.22	46.34	9.44	1791

2023	0–39	67.65	27.99	4.36	4151
2023	40–59	69.11	28.07	2.82	7721
2023	60+	59.21	37.04	3.76	1917

**Table 3:** Chi-square results between 2019 and 2021.

City	Gender	Age range	*p* value
Berlin	M	0–39	9.59*e* − 06
Berlin	F	0–39	4.73*e* − 03
Berlin	M	40–59	1.31*e* − 07
Berlin	F	40–59	2.89*e* − 04
Berlin	M	60+	2.44*e* − 09
Berlin	F	60+	5.83*e* − 13

Chicago	M	0–39	5.79*e* − 08
Chicago	F	0–39	2.25*e* − 03
Chicago	M	40–59	1.99*e* − 09
Chicago	F	40–59	1.63*e* − 04
Chicago	M	60+	4.08*e* − 05
Chicago	F	60+	3.37*e* − 04

London	F	60+	0.04

**Table 4:** Wasserstein distance results.

City	Age range	Gender	2018–2019	2019–2021	2021–2022	2022–2023
Boston	0–39	M	4.49	2.06	5.89	7.94
Boston^∗†^	0–39	F	2.62	4.3	10.05	8.19
Boston^†^	40–59	M	4.15	6.36	7.09	9.45
Boston^†^	40–59	F	4.14	5.23	5.94	8.24
Boston^†^	60+	M	2.22	6.18	8.52	8.58
Boston^∗†^	60+	F	0.88	2.2	10.93	6.85

Berlin^†^	0–39	M	9.18	8.4	6.05	1.23
Berlin^∗†^	0–39	F	7.07	10.82	9.56	1.26
Berlin^†^	40–59	M	10.46	6.12	3.88	0.44
Berlin^∗†^	40–59	F	6.91	9.53	5.57	0.31
Berlin^∗†^	60+	M	4.7	10.67	12.54	1.07
Berlin^∗†^	60+	F	5.31	6.48	12.94	0.63

Chicago^∗†^	0–39	M	3.8	8.53	7.6	4.74
Chicago^†^	0–39	F	1.34	1.96	3.23	5.59
Chicago^∗†^	40–59	M	2.66	9.59	9.08	5.36
Chicago^†^	40–59	F	1.45	3.78	4.37	6.5
Chicago^∗†^	60+	M	1.03	12.39	12.34	4.44
Chicago^∗†^	60+	F	0.21	6.21	7.46	6.23

London	0–39	M	7.52	0.7	1.8	6.2
London	0–39	F	13.5	1.89	0.69	0.7
London	40–59	M	15.94	2.48	0.77	3.67
London	40–59	F	14.14	4.16	1.29	2.02
London	60+	M	13.21	5.14	0.74	6.37
London	60+	F	19.54	9.78	1.98	7.62

Paris	0–39	M	6.95	0.69	0.69	5.4
Paris	0–39	F	3.08	0.59	0.18	5.51
Paris	40–59	M	6.45	3.68	1.69	5.67
Paris	40–59	F	2.28	4.64	2.17	6.68
Paris	60+	M	15.17	5.37	2.69	6.75
Paris^†^	60+	F	11.7	3.86	2.24	1.97

Valencia	0–39	M	2.76	1.7	2.81	2.94
Valencia^∗†^	0–39	F	1.11	3.17	4.79	0.73
Valencia^∗†^	40–59	M	1.72	1.85	3.59	2.54
Valencia^∗†^	40–59	F	2.54	0.88	3.82	0.4
Valencia^∗†^	60+	M	2.61	5.38	8.23	3.45
Valencia^∗†^	60+	F	1.43	3.48	5.75	2.5

^∗^Means that either distance 2019–2021 or 2021–2022 is the maximum of the four calculated distances.

^†^Means that either distance 2018–2019 or 2022–2023 is the minimum.

**Table 5:** Wasserstein distance ranking results.

City	Age range	Gender	Higher distance	2nd higher distance	3rd higher distance
Berlin	0–39	F	19–21	21–22	18–19
Berlin	60+	M	21–22	19–21	18–19
Berlin	60+	F	21–22	19–21	18–19

Chicago	0–39	M	19–21	21–22	22–23
Chicago	40–59	M	19–21	21–22	22–23
Chicago	60+	M	19–21	21–22	22–23

Valencia	0–39	F	21–22	19–21	18–19
Valencia	60+	M	21–22	19–21	22–23
Valencia	60+	F	21–22	19–21	22–23

Boston	0–39	F	22–23	21–22	19–21
Boston	40–59	M	22–23	21–22	19–21
Boston	40–59	F	22–23	21–22	19–21
Boston	60+	M	22–23	21–22	19–21
Boston	60+	F	21–22	22–23	19–21

Berlin	0–39	M	18–19	19–21	21–22
Berlin	40–59	M	18–19	19–21	21–22
Berlin	40–59	F	19–21	18–19	21–22

Chicago	0–39	F	22–23	21–22	19–21
Chicago	40–59	F	22–23	21–22	19–21
Chicago	60+	F	21–22	22–23	19–21

Paris	60+	F	18–19	19–21	21–22

Valencia	40–59	M	21–22	22–23	19–21
Valencia	40–59	F	21–22	18–19	19–21

*Note:* The first 9 elements are groups with the highest distances in 2019–2021 or 2021–2022. All the elements in the table are groups with the three highest distances in 2019–2021 or 2021–2022.

## Data Availability

The data that support the findings of this study are openly available in the following websites: • BMW BERLIN-MARATHON; 2025; Results and certificates; https://www.bmw-berlin-marathon.com/en/your-race/results • BOSTON MARATHON; 2025; RESULTS. https://www.baa.org/races/boston-marathon/results/search-results • Bank of America Chicago Marathon; 2025; Race Results. https://chicago-history.r.mikatiming.com/2023/ • TCS London Marathon; 2025; Tracking & Results 2025. https://results.tcslondonmarathon.com/2025/ • Schneider Electric Marathon de Paris; 2025; Results. https://www.schneiderelectricparismarathon.com/en/event/results • Valencia Marathon Trinidad Alfonso; 2025; Previous editions. https://www.valenciaciudaddelrunning.com/en/marathon/previous-editions-marathon/

## Endnotes

^1^Hottest London Marathon (https://www.bbc.com/news/uk-england-london-43850037).

^2^Racecast.io Paris Marathon (https://racecast.io/race/paris-marathon).
